# Animal Heat as Affected by Phthisis

**Published:** 1848-01

**Authors:** Charles Scudamore


*Animal Heat as affected by Phthisis.* By SIR CHARLES SCUDAMORE.—"It is a curious pathological fact, as I have found in a very extensive examination to be verified without a single exception, that in every case of tubercular phthisis the animal heat is more or less raised beyond the healthy standard. This may be stated as a mean 96·5. It is always found highest in the morning at the time of rising from bed, with all persons. In the course of the day it is influenced by certain circumstances, and is raised by exercise; particularly in the fresh air of the country. In phthisis, I have found it range from 99° to 105°. I consider that the examination of the animal heat assists our diagnosis as to the existence of tubercles. In a doubtful case, I am pleased to find the animal heat not higher than 98°.

"It would be foreign to my present purpose to enter into the interesting, beautiful, yet difficult subject of animal heat; or attempt the consideration how far this function is to be referred to chemical action taking place in the lungs, how far to vital influence, and how far to the nervous system; but I believe it is on all hands agreed that the most immediate and influential cause of the production of animal heat is the combustion of carbon, brought in the venous blood to the ramifying capillaries, in order to receive aeration and the all-important influence of oxygen introduced by the air-passages.

"In this chemical view of the subject, and to which I now confine myself, it appears, I think, surprising, when we consider how much of tuberculated lungs is excluded from the aerating process, either from the compression of the air-cells by the tubercles, or their actual occupation by these foreign bodies, that the animal heat, instead of being lower, as we might imagine, as a consequence of the surface for the aeration of the blood being greatly lessened, is actually found to be higher than in the natural healthy state of the lungs.

After relating some experiments which go to show that the quantity of carbonic acid evolved during respiration is greater in a phthisical patient than in a healthy person, our author remarks:—

"It appears to me that, in the relation of these experiments, I have offered strong evidence that in tubercular phthisis, notwithstanding the organic limitation of the function of the lungs, from the obstruction of the air-cells by tubercles, the important process of the decarbonisation of the blood, the combustion of carbon, and increased animal heat beyond the healthy standard, go on with more rapidity than in sound lungs. An increase of the animal heat does not ensue from merely quickened respiration, as is shown in the examples of running up to the top of a lofty building; nor even more than a degree in the paroxysm of spasmodic asthma. I consider, indeed, that in some cases of this kind, where almost asphyxia takes place, the reverse would happen, and the animal heat be found below the natural standard.

"There is in tuberculated lungs an increased activity of function; and hence the hectic fever, so urgent in acute phthisis, and the hectic irritation, although often hardly amounting to evident fever, in the chronic form of the disease. The actions of the animal economy, when unnaturally hurried, are not so healthily performed. We may suppose that the oxidation of the blood being effected in so rapid a manner cannot be so favorably accomplished; and also a morbid excitement prevails in the whole system. The nervous system is morbidly sensitive. Although the appetite may be good, and abundance of food be taken, yet nutrition is imperfect, and the body wastes—a consequence much to be referred to the imperfect and unhealthy performance of assimilation and sanguification; at the same time that the absorbants generally are probably thrown into a state of morbid activity."—*Medico-Chirurgical Review.*

